# Caffeic Acid Phenethyl Ester-Incorporated Radio-Sensitive Nanoparticles of Phenylboronic Acid Pinacol Ester-Conjugated Hyaluronic Acid for Application in Radioprotection

**DOI:** 10.3390/ijms22126347

**Published:** 2021-06-14

**Authors:** Seon-Hee Choi, Dong-Yeon Lee, Sohi Kang, Min-Kyung Lee, Jae-Heun Lee, Sang-Heon Lee, Hye-Lim Lee, Hyo-Young Lee, Young-IL Jeong

**Affiliations:** 1 Biomedical R&D Center, Pusan National University Yangsan Hospital, Gyeongnam 50612, Korea; jsmsmo@naver.com (S.-H.C.); roasua@hanmail.net (H.-L.L.); 2 Department of Radiation Oncology, Dongnam Institute of Radiological & Medical Sciences, Pusan 46033, Korea; gymnist@dirams.re.kr; 3 Biomaterial R&BD Center, Chonnam National University, Gwangju 61186, Korea; shrloveu@gmail.com; 4 Department of Dental Hygiene, Dong-Eui University, Pusan 47340, Korea; lmk849@deu.ac.kr; 5 Department of Radiological Science, Dong-Eui University, Pusan 47340, Korea; dudrkarka@naver.com (J.-H.L.); heon2k@naver.com (S.-H.L.)

**Keywords:** radioprotection, caffeic acid phenetyl ester, reactive oxygen species, phenylboronic acid, radiosensitive nanoparticles

## Abstract

We synthesized phenylboronic acid pinacol ester (PBPE)-conjugated hyaluronic acid (HA) via thiobis(ethylamine) (TbEA) linkage (abbreviated as HAsPBPE conjugates) to fabricate the radiosensitive delivery of caffeic acid phenetyl ester (CAPE) and for application in radioprotection. PBPE was primarily conjugated with TbEA and then PBPE-TbEA conjugates were conjugated again with hyaluronic acid using carbodiimide chemistry. CAPE-incorporated nanoparticles of HAsPBPE were fabricated by the nanoprecipitation method and then the organic solvent was removed by dialysis. CAPE-incorporated HAsPBPE nanoparticles have a small particle size of about 80 or 100 nm and they have a spherical shape. When CAPE-incorporated HAsPBPE nanoparticles were irradiated, nanoparticles became swelled or disintegrated and their morphologies were changed. Furthermore, the CAPE release rate from HAsPBPE nanoparticles were increased according to the radiation dose, indicating that CAPE-incorporated HAsPBPE nanoparticles have radio-sensitivity. CAPE and CAPE-incorporated HAsPBPE nanoparticles appropriately prevented radiation-induced cell death and suppressed intracellular accumulation of reactive oxygen species (ROS). CAPE and CAPE-incorporated HAsPBPE nanoparticles efficiently improved survivability of mice from radiation-induced death and reduced apoptotic cell death. We suggest that HAsPBPE nanoparticles are promising candidates for the radio-sensitive delivery of CAPE.

## 1. Introduction

Ionizing radiation has been extensively used for therapeutic and diagnostic purposes of human disease [1,2,3,4,5]. Radiation therapy is regarded as one of the primary treatment options for cancer patients since it is a promising candidate for inoperable cancer and induces apoptosis/necrosis of cancer cells [1,2,3]. Furthermore, ionizing radiation has been extensively used for medical imaging of human disease since it is still considered as an ideal tool for detection of disease and malignancies [4,5]. Even though ionizing radiation is widely utilized for diagnosis/therapy of human diseases, repeated or high dose radiation is closely associated with undesirable effects against healthy cells and tissues since ionizing radiation induces oxidative stress in the surrounding field of irradiation [6,7,8]. Irradiation causes DNA damage and metabolic stress in healthy cells and tissues since it elevates the ROS level not only in disease cells/tissues but also in healthy cells/tissues [7,8]. To overcome these drawbacks, many scientists have investigated the strategy for radioprotection of healthy cells/tissues from irradiation [7,8,9,10]. For example, Liu et al. reported that whey hydrolysate peptides have effectiveness in radioprotection of mice from ^60^Coγ radiation damage and then prolonged survival time of mice [7]. Otherwise, amifostine is widely used in clinics to attenuate radiation-induced damage [11,12]. However, the side effects of amifostine are also problematic such as severe skin eruption, hypotension, fever/rash, immune hypersensitivity syndrome, and fatigue, etc. [13,14,15,16]. For these reasons, antioxidants efficiently attenuate tissue injury from radiation-induced damage [9,10]. Qin et al. reported that resveratrol inhibits oxidative stress from radiation and then reduces intestinal injury [9]. Epigallocatechin gallate (EGCG) is known to decrease ROS levels, suppress radiation-induced intestinal injury, and then prolong the survival time of mice [10].

Caffeic acid phenethyl ester (CAPE), which is a propolis of honeybee hives, has various biological activities such as antioxidants, anti-allergenic, anticarcinogenic, anti-inflammatory, and immunomodulation [17,18,19,20,21,22]. CAPE has an inhibitory effect against nuclear oxygenation of linoleic acid and arachidonic acid and an effectiveness in the decrease of chemotherapy or radiation therapy-induced damages [17]. Furthermore, CAPE was considered as a strong antioxidant in the biological system [23,24,25]. Nasution et al. reported the inhibitory effect against oxidative stress in brain injury [23]. CAPE also has a protective effect against nephrotoxicity and oxidative kidney damage [24]. CAPE showed an anti-oxidative effect against the experimental periodontitis model, i.e., it decreases the total oxidant status in gingival tissues and then improves therapeutic potential [25]. Especially, the pretreatment of CAPE prior to irradiation of rats effectively protects radiation-induced hepatic or brain injury [26,27]. Chu et al. reported that CAPE administration decreased the serum levels of alanine aminotransferase and aspartate aminotransferase, while the activity of superoxide dismutase and glutathione was increased [26]. Then, CAPE effectively protected from radiation-induced hepatotoxicity and inhibited hepatocyte apoptosis. The pretreatment of CAPE also protects brain damage from ionizing radiation through the control of total oxidant status and oxidative stress index [27].

Nano-dimensional vehicles such as nanoparticles, polymeric micelles, liposomes or colloidal carriers have been extensively investigated in the diverse field of biomedical science [28,29,30,31,32]. Especially, nanoparticles have been frequently considered for stimuli-sensitive drug delivery of bioactive agents, i.e., they can be modified to respond against the stimulus in the biological system such as acidic pH, temperature, magnetic field, oxidative stress, light, and irradiation [32,33,34,35,36,37]. For example, Choi et al. reported that iron oxide-incorporated lipocomplexes respond to the magnetic field, concentrate around the tumor mass, and then specifically target cancer [32]. Polymer conjugates having diselenide linkage can be used to target cancer cells and deliver the cancer drug through reactive oxygen species (ROS)-sensitive cleavage of selenide linkage since the tumor microenvironment normally revealed increased redox potential and elevated ROS level [35]. Choi et al. reported that methoxy poly(ethylene glycol)-b-poly(DL-lactide-co-glycolide) block copolymer nanoparticles having diselenide linkages responded to irradiation and then specifically released ebselen, i.e., they showed that the ebselen release rate was accelerated according to the dose of irradiation [37]. Furthermore, the antibody decorated lipid nanoparticles incorporating CAPE are preferentially target disease cells with low intrinsic cytotoxicity against normal cells [38]. Nanoparticles are reasonable candidates for the delivery of bioactive agents to improve biological activity and to reduce genotoxicity [38,39,40].

In this study, we synthesized the HAsPBPE copolymer via sulfide linkage to fabricate nanoparticles for ROS-sensitive and/or radio-sensitive delivery of CAPE. Since CAPE is a lipophilic chemical, nanoparticles can be used as an ideal carrier for parenteral injection of CAPE and improve its biological activity in vitro and in vivo.

## 2. Results

### 2.1. Synthesis of HAsPBPE Conjugates

**Figure 1:**
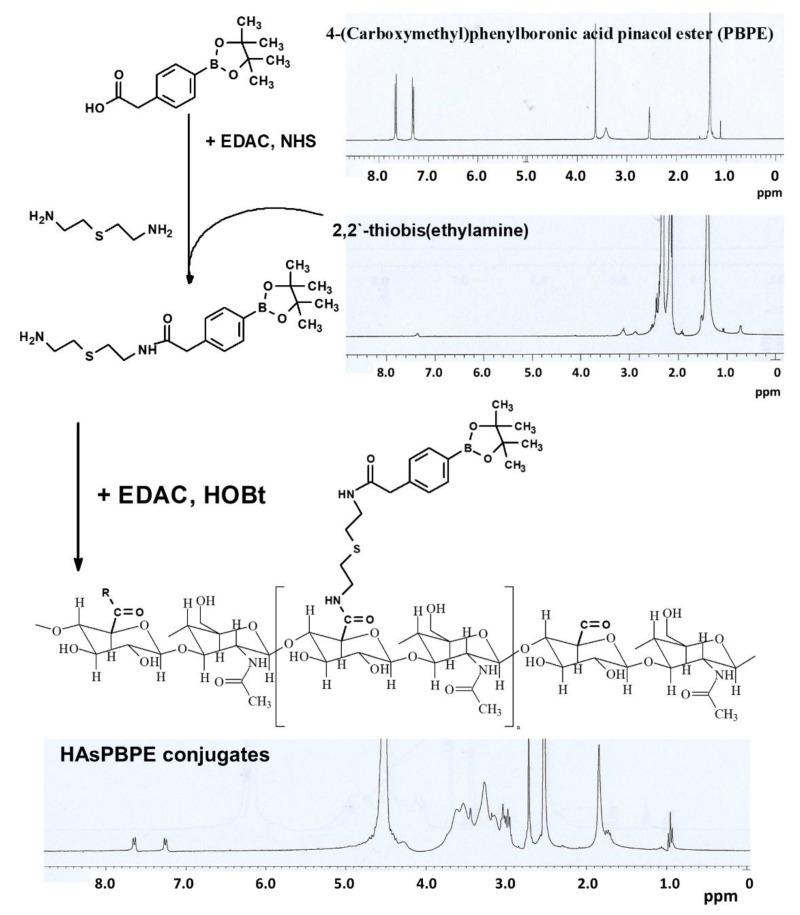
Synthesis scheme and ^1^H NMR spectra of HAsPBPE conjugates.

Figure 1 shows the synthesis scheme and ^1^H NMR spectra of each chemical. The carboxyl group of PBPE was primarily conjugated with the amine group of TbEA and then PBPE-TbEA conjugates were conjugated with the carboxyl group of HA (HAsPBPE conjugates). As shown in Figure 1, PBPE itself showed an intrinsic peak between 1.0 and 8.0, while TbEA also showed an intrinsic peak between 1.0 and 3.4. As shown in Figure 1, 1H NMR spectra of HAsPBPE shows specific peaks of PBPE between 7.0~8.0 and TbEA between 1.0~2.8, while specific peaks of HA were confirmed at 2.9~4.0.

**Figure 2:**
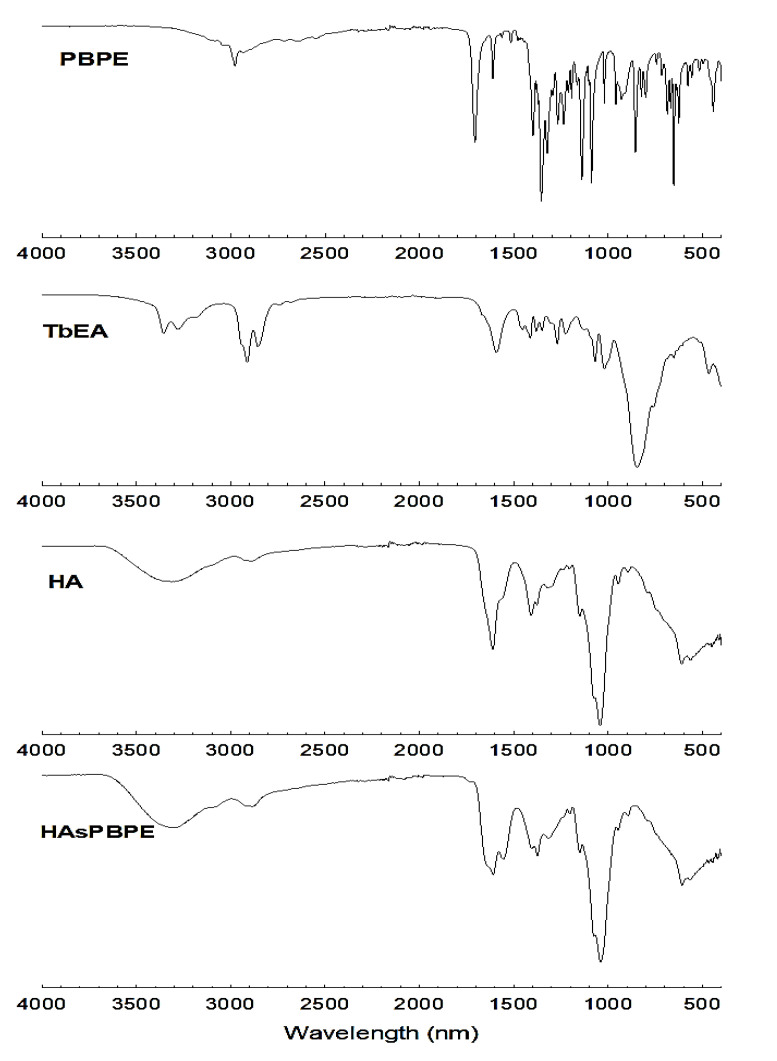
FT-IR spectra of PBPE, TbEA, HA, and HAsPBPE conjugates.

Figure 2 shows the FT-IR spectra of each chemical. As shown in Figure 2, the C=O stretch of PBPE was confirmed at about 1720 cm^−1^, while the primary amine of TbEA was confirmed at about 1619 cm^−1^. The peptide bond (O=C–NH) of HAsPBPE from the conjugation between the carboxyl group of HA and amine group of PBPE-TbEA conjugates were confirmed at 1550 and 1650 cm^−1^. These results show that HAsPBPE was successfully synthesized.

### 2.2. Preparation and Characterization of CAPE-Incorporated HAsPBPE Nanoparticles

**Figure 3:**
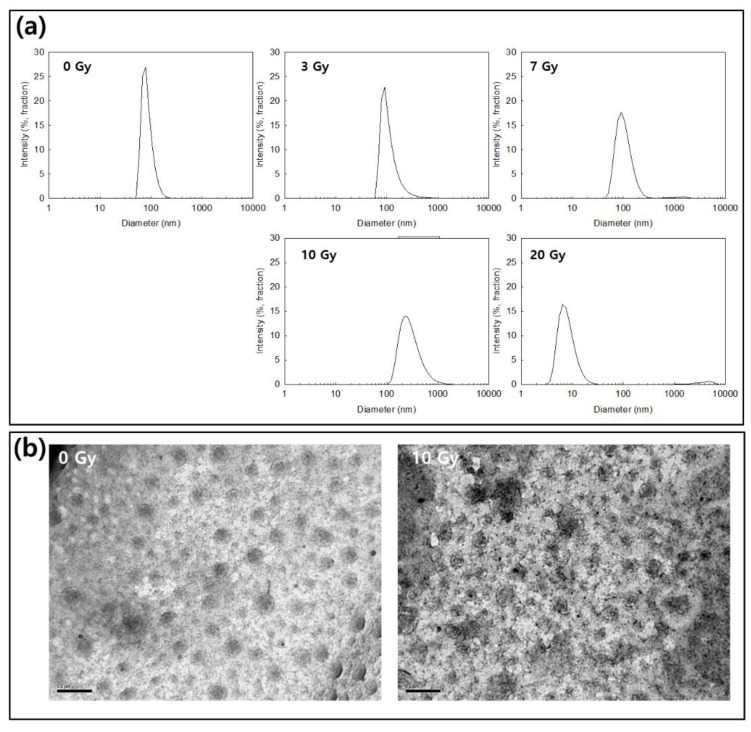
(**a**) The effect of irradiation on the changes of the particle size of CAPE-incorportaed HAsPBPE nanoparticles. (**b**) The effect of irradiation on the morphological changes of CAPE-incorportaed HAsPBPE nanoparticles (bar = 200 nm).

CAPE-incorporated HAsPBPE nanoparticles were prepared by the nano-precipitation method and then the organic solvent was removed by dialysis. HAsPBPE nanoparticles showed a small diameter less than 70 nm as abbreviated in Table 1. Particle sizes were gradually increased according to the increase of drug contents. When feeding of the drug in the nanoparticle preparation process was increased, drug contents were also increased, i.e., drug contents were increased 7.5 (*w*/*w*) and 12.3% (*w*/*w*). Figure 3 shows the changes of particle size distribution and morphology of CAPE-incorporated HAsPBPE nanoparticles. Practically, CAPE-incorporated HAsPBPE nanoparticles have a spherical shape and their particle size showed mono-modal distribution (0 Gy in Figure 3a,b). However, particle sizes were gradually increased and became a broad distribution by irradiation, i.e., the size distribution became broader than 100 nm by irradiation at 7 Gy and then nanoparticles seem to be disintegrated at 20 Gy irradiation (Figure 3a). Figure 3b shows that nanoparticles were disintegrated by 20 Gy irradiation, while nanoparticles at 0 Gy revealed spherical shapes. Practically, the particle size was not properly measured by irradiation with 20 Gy as shown in Figure 3a. These results indicated that physicochemical properties of HAsPBPE nanoparticles can be changed and their morphologies were disintegrated by irradiation.

**Figure 4:**
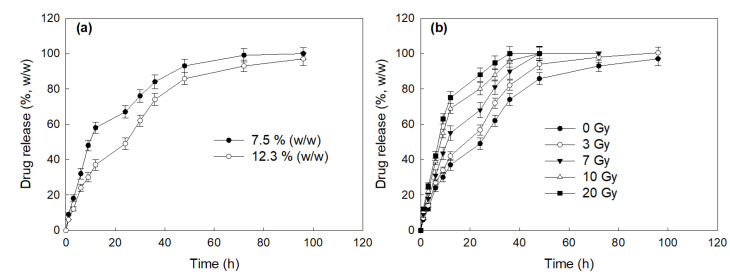
(**a**) The effect of drug contents on the drug release from HAsPBPE nanoparticles. (**b**) The effect of irradiation on the changes of the particle size of CAPE-incorportaed HAsPBPE nanoparticles.

Figure 4 shows the drug release behavior of HAsPBPE nanoparticles. As shown in Figure 4a, the higher drug contents induced the delayed release of the drug from the nanoparticles. The burst release behavior was observed until 12 h and then the drug was continuously released until 4 days. These results might be due to the fact that the CAPE existing near the surface was released rapidly and then resulted in the burst release behavior of nanoparticles. Interestingly, the drug release was gradually increased according to the increase of irradiation dose, i.e., the drug release rate was accelerated by the increased dose of irradiation as shown in Figure 4b. These results indicated that HAsPBPE nanoparticles respond to irradiation and then they can be disintegrated or swelled in an aqueous solution. These behaviors induced the acceleration of drug release from nanoparticles.

### 2.3. Biological Activity of CAPE-Incorporated HAsPBPE Nanoparticles

**Figure 5:**
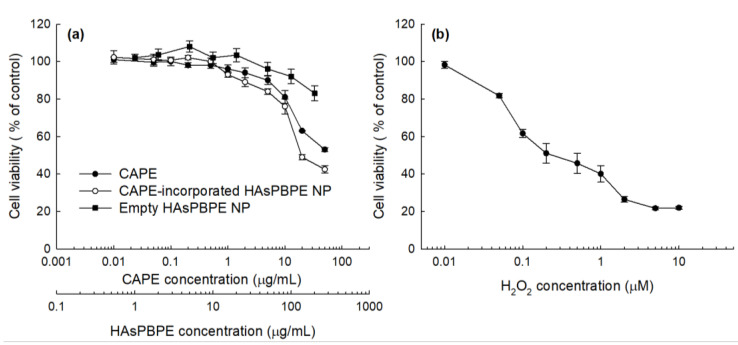
The effect of CAPE or CAPE-incorporated HAsPBPE nanoparticles (**a**) and H_2_O_2_ (**b**) on the viability of L929 cells.

Figure 5 showed the viability of L929 cells against CAPE, CAPE-incorporated HAsPBPE nanoparticles, and H_2_O_2_. As shown in Figure 5a, the viability of L929 cells were dose-dependently decreased according to the CAPE concentration. However, cell viability at lower than 5 μg/mL of CAPE concentration was higher than 80% of both free CAPE and CAPE-incorporated nanoparticles, i.e., the treatment with CAPE-incorporated HAsPBPE nanoparticles showed 84% cell viability at 5 μg/mL and 76% at 10 μg/mL. The free CAPE treatment also resulted in higher than 90% at 5 μg/mL and 81% at 10 μg/mL. The treatment with empty HAsPBPE nanoparticles maintained higher than 80% cell viability at 200 μg/mL. CAPE-incorporated nanoparticles were slightly cytotoxic compared to CAPE itself as shown in Figure 5a. These results might be due to the fact that the viability of cells was slightly decreased at higher than 100 μg/mL of the empty HAsPBPE concentration even though viability was still maintained at higher than 80% at 100 μg/mL of the empty HAsPBPE concentration. Then, HAsPBPE conjugates may affect the viability of cells. When L929 cells were exposed to H_2_O_2_, the viability of cells were gradually decreased according to the concentration of H_2_O_2_ (Figure 5b).

**Figure 6:**
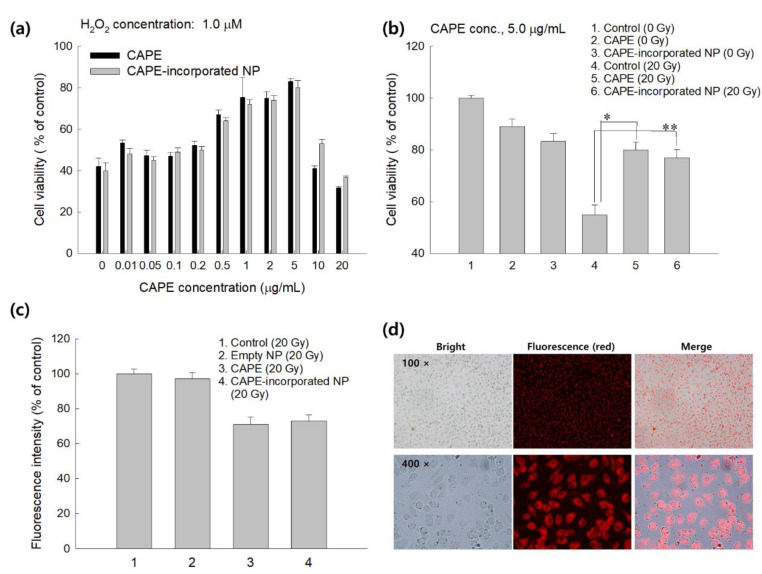
(**a**) The effect of CAPE or CAPE-incorporated HAsPBPE nanoparticles on the viability of L929 cells in the presence of H_2_O_2_. (**b**) The effect of irradiation on the viability of L929 cells. (**c**) Intracellular ROS level pretreated with CAPE or CAPE-incorporated HAsPBPE nanoparticles and irradiation. Cell were exposed to 20 Gy radiation. (**d**) Fluorescence observation of cellular uptake of Ce6-incorporated HAsPBPE nanoparticles. Ce6-incorporated HAsPBPE nanoparticles (final concentration of Ce6: 2 μg/mL. Cells were treated with nanoparticles for 2 h). *, **: *p* < 0.01.

To study the protective effect against oxidative-stress, CAPE and CAPE-incorporated nanoparticles were treated to L929 cells and then exposed to H_2_O_2_ as shown in Figure 6. As shown in Figure 6a, the viability of L929 cells were less than 50% when they were exposed to H_2_O_2_. However, their viability was gradually increased by the pretreatment with CAPE or CAPE-incorporated nanoparticles until 5 μg/mL of CAPE concentration. No differences between CAPE and CAPE-incorporated nanoparticles were obtained. Practically, the protective effect against oxidative stress was not observed at higher than 10 μg/mL of CAPE concentration. These results might be due to the cytotoxicity of CAPE. When L929 cells were irradiated at 20 Gy, the viability of cells was significantly decreased by less than 70%. However, the viability of cells was increased over 20% when cells were pretreated with CAPE or CAPE-incorporated nanoparticles. These results indicated that CAPE-incorporated nanoparticles properly protect cells from oxidative stress and irradiation as well as CAPE itself. Figure 6c shows ROS generation by irradiation of L929 cells and then the scavenging effect of CAPE or CAPE-incorporated nanoparticles. As shown in Figure 6c, irradiation of cells induces ROS generation from cells. However, cells pretreated with CAPE or CAPE-incorporated nanoparticles properly suppressed the intracellular ROS level by irradiation of cells, while empty HAsPBPE nanoparticles themselves did not affect the intracellular ROS level. These results indicated that CAPE or CAPE-incorporated nanoparticles have a protective effect against oxidative stress and irradiation against cells.

### 2.4. In Vivo Animal Study

**Figure 7:**
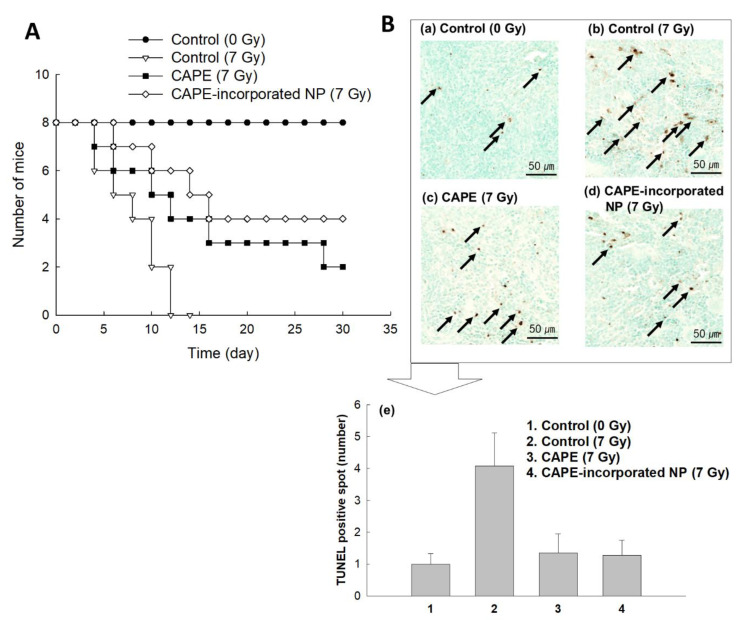
(**A**) The effect of CAPE or CAPE-incorporated HAsPBPE nanoparticles on the survivability of mice**.** BALb/C mice were i.v. injected CAPE or CAPE-incorporated HAsPBPE nanoparticles 1 h before irradiation. The CAPE dose was 10 mg/kg. (**B**) TUNEL staining of spleen sections. TUNEL staining of spleen sections demonstrated the protective effect of CAPE or CAPE-incorporated HAsPBPE nanoparticles after irradiation. (**a**) Control group (0 Gy); (**b**) control (7 Gy): The spleen histology of the irradiation group showed decreased TUNEL-positive cells (black arrow); (**c**) CAPE (7 Gy); (**d**) CAPE-incorporated HAsPBPE nanoparticles group (7 Gy). (**e**) Changes of TUNEL-positive staining of apoptotic cells (dark spot). Average ± SD from ten images. Apoptotic spot was calculated from ten captured areas as shown in Figures S1 and S2. Statistical analysis: 1:3, 1:4, *p* < 0.15; 2:3, 2:4, *p* < 0.1.

To assess the protective effect against irradiation, CAPE or CAPE-incorporated nanoparticles were i.v. administered via the tail vein of mice and then their whole body was irradiated. Survivability of mice was monitored over 1 month as shown in Figure 7. When mice were irradiated, all of the mice died before 12 days due to the irradiation as shown in Figure 7A. However, more than four mice treated with CAPE or CAPE-incorporated nanoparticles were survived until 2 weeks. Especially, four mice treated with CAPE-incorporated nanoparticles were survived over 4 weeks. These results indicated that CAPE-incorporated nanoparticles have a superior activity against irradiation. As shown in Figure 7B, TUNEL staining of the spleen section supported these results. TUNEL staining of the spleen section revealed many apoptotic cells in the spleen section when mice were irradiated. CAPE or CAPE-incorporated nanoparticles significantly decrease the apoptotic cell, indicating that they properly protect cells from irradiation. To count the TUNEL-positive spot as an apoptotic cell, ten areas (300 × 300 m) in tissue sections of the spleen were randomly selected and counted as shown in Figures S1 and S2. Figure 7B(e) shows that CAPE or CAPE-incorporated nanoparticles decreased apoptotic cell death, indicating that CAPE and CAPE-incorporated nanoparticles have a radioprotective effect in the biological system.

**Figure 8:**
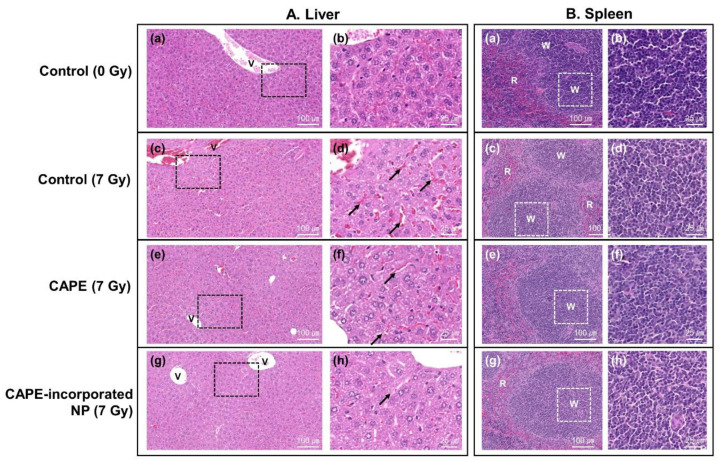
(**A**) H&E staining of mouse liver. Photomicrographs of liver sections stained with H&E demonstrated the protective effective of CAPE or CAPE-incorporated HAsPBPE nanoparticles when the whole body of the mouse was exposed to irradiation. (**a**,**b**) Control group (0 Gy); (**c**,**d**) control (7 Gy): The liver histology of the irradiation group showed a hepatocyte edema and sinusoidal dilation (d, black arrow); (**e**,**f**) CAPE (7 Gy); (**g**,**h**) CAPE-incorporated HAsPBPE nanoparticles group (7 Gy). (**B**) H&E staining of mouse spleen**.** Photomicrographs of spleen sections stained with H&E demonstrated the protective effective of CAPE with empty HAsPBPE nanoparticles or CAPE-incorporated HAsPBPE nanoparticles when the whole body of the mouse was exposed to irradiation. (**a**,**b**) Control group; (**c**,**d**) irradiation-only group; the spleen histology of the irradiation group showed a decrease in the lymphocytes in the white pulp; (**e**,**f**) CAPE + empty nanoparticles group; (**g**,**h**) CAPE-incorporated HAsPBPE nanoparticles group. Arrows, dilated sinusoid, exudate red blood cells (RBC); v, hepatic vein; W, white pulp; R, red pulp.

Figure 8 showed the H&E staining of liver (Figure 8A) and spleen (Figure 8B). As shown in Figure 8A, the control group revealed normal cellular characteristics of liver, including normal parenchyma, unremarkable hepatocytes, canaliculi, and canals of liver histology. The liver histology of the irradiation group (control (7 Gy)), however, showed extensive hepatocellular edema and sinusoidal dilation (with dilated endothelial cells). The liver histology of mice treated with CAPE or CAPE-incorporated nanoparticles with irradiation showed definitely better cellular architecture than that of the irradiation group (control (7 Gy)). The spleen histological sections of the control group (control (0 Gy)) revealed normal cellular characteristics with red and white pulp, as shown in Figure 8B. Whereas the spleen histology of the irradiation group (control (7 Gy)) indicated a decrease in lymphocytes in the white pulp, it was clear that the pretreatment with CAPE or CAPE-incorporated nanoparticles certainly revealed decreased radiation-induced damage in the spleens and improved spleen morphology. These results indicated that CAPE-incorporated nanoparticles as well as CAPE definitely protected mice from the irradiation-induced damage and then improved survivability of mice.

## 3. Discussion

Even though ionizing irradiation has been extensively used for diagnosis and therapeutic purposes, risks from radiation damage are always problematic in the human body. Especially, it is known that the ROS level is significantly increased in the field of irradiation and then these induce oxidative stress against cells and tissues [6,41]. The increased ROS and/or reactive nitrogen species (RNS) level induce metabolic stress and affect the nuclear function of DNA repair mechanism [41]. Due to these reasons, clinicians use a radioprotectant such as amifostine to prevent radiation-induced cell/tissue damage [11,12]. However, many scientists have tried to develop novel and non-toxic antioxidants since adverse side effects of amifostine are always problematic in clinical use [13,14,15,16]. Many kinds of antioxidants have been investigated to prevent radiation-damage and to substitute amifostine [7,8,9,10,14,15,42,43]. For example, Bai et al. reported that CAPE effectively prevents cellular oxidative stress from radiation by elimination of free radicals and then improves cell viability [42]. They argued that CAPE properly reduced the intracellular ROS level after radiation of L929 cells. Song et al. also reported that intracellular ROS accumulation induced by H_2_O_2_ stimulation was effectively inhibited by the pretreatment of CAPE [44]. They argued that H_2_O_2_-induced upregulation of TNF-α and COX-2 expression can be suppressed by the treatment of CAPE with a dose/time dependent manner. Moreover, we showed that CAPE-incorporated nanoparticles as well as CAPE effectively suppressed intracellular ROS accumulation induced by the irradiation of cells. These behaviors must be correlated with higher viability of cells as shown in Figure 6b,c. Furthermore, CAPE and CAPE-incorporated nanoparticles efficiently prevented H_2_O_2_-induced cell death, i.e., the viability of cells treated with CAPE or CAPE-incorporated nanoparticles was almost two times higher than the control (Figure 6a). CAPE or CAPE-incorporated nanoparticles against H_2_O_2_-induced cell death were dose-dependently protected cells. Furthermore, they also showed positive effects against survivability of mice from irradiation. Especially, CAPE-incorporated nanoparticles showed extended survivability of mice after irradiation at 7 Gy compared to CAPE itself (Figure 7). These results indicated that CAPE-incorporated nanoparticles have a superior activity against radiation-induced injury. Actually, CAPE-incorporated nanoparticles appropriately reduced apoptotic cell death and improved tissue morphologies as shown in Figure 8. CAPE and CAPE-incorporated nanoparticles are a great potential for preventing radiation-induced injury. Yoncheva et al. reported that CAPE-incorporated polymeric micelles exhibited a superior protective effect against H_2_O_2_-induced oxidative stress compared to the free CAPE treatment [39]. Furthermore, CAPE-loaded poly(DL-lactide-co-glycolide) nanoparticles are more effective in the reduction of mutagenicity than the free CAPE itself [40]. We also prepared CAPE-incorporated nanoparticles using HAsPBPE conjugates since HA is a biocompatible and water-soluble biopolymer, and TbEA-PBPE moiety acts as a hydrophobic moiety in polymer backbone [45]. Then, HAsPBPE conjugates may form core-shell type nanoparticles, i.e., HA acts as an outershell of the nanoparticles while TbEA-PBPE is composed of the hydrophobic inner-core of the nanoparticles. Nanoparticles showed spherical morphologies with small particle sizes less than 100 nm in the average diameter as shown in Figure 3. In the drug release experiment, nanoparticles showed a burst release behavior until 12 h and then was released continuously until 96 h, indicating that the CAPE existing near the surface of the nanoparticles was released rapidly and, after that, the CAPE in the core of the nanoparticles was released more slowly. In our previous reports, the burst release of CAPE from nanoparticles was also observed in the initial phase of drug release study followed by a sustained release pattern [46].

Nanoparticulate-based delivery systems for bioactive agents are suitable for eliminating disease cells such as cancer stem cells and ROS [47,48]. For example, polymeric nanoparticles decorated with specific ligands or antibodies have the potential to target and reject cancer stem cells [47]. Furthermore, nanocarriers incorporating the dual or multi drug delivery strategy have a great potential to diminish ROS production in disease cells and then reduce oxidative damage in mitochondria [48]. Then, these dual/multi drug deliveries using nanocarriers may provide a synergistic effect in the improvement of therapeutic index of neuroregenerative strategy [48]. Our concept for radioprotection is to develop radio-sensitive nanomedicine for the sustained and/or radio-responsive release of CAPE. Since CAPE itself is a lipophilic agent and is hardly dissolved in an aqueous solution, nanoparticles can solve these problems, i.e., nanoparticles enable us to dissolve CAPE in an aqueous solution [28]. Furthermore, nanoparticles can be modified to be sensitive to physicochemical stimulus such as radiation, pH, ROS, magnetic field, and light [32,33,34,35,36,37]. HAsPBPE nanoparticles can respond to ROS since the PBPE moiety and thiol group in TbEA in the polymer backbone are known to have ROS-sensitivity and these moieties are able to be decomposed in the oxidative stress [45].

Actually, CAPE-incorporated nanoparticles of HAsPBPE were disintegrated in the presence of H_2_O_2_ as shown in Figure 3a,b. HAsPBPE nanoparticles showed an accelerated CAPE release through irradiation (Figure 4). Then, HAsPBPE nanoparticles specifically decomposed and released CAPE when they were exposed to irradiation, i.e., drug release can be suppressed in the absence of irradiation and then irradiation of nanoparticles accelerated drug release. Even though nanoparticles in the in vitro cell culture showed a similar protective activity compared to CAPE, they were improved by the biological activity in the in vivo animal irradiation study, as shown in Figure 7 and Figure 8.

In conclusion, we synthesized HAsPBPE conjugates to fabricate CAPE-incorporated nanoparticles and applied them in radioprotection of cells. HAsPBPE nanoparticles showed changes in particles according to the irradiation dose and their morphologies were disintegrated by irradiation. Furthermore, the CAPE release rate from HAsPBPE nanoparticles were increased according to the radiation dose, indicating that CAPE-incorporated HAsPBPE nanoparticles have radio-sensitivity. CAPE and CAPE-incorporated HAsPBPE nanoparticles appropriately prevented radiation-induced cell death and suppressed intracellular ROS accumulation. CAPE and CAPE-incorporated HAsPBPE nanoparticles efficiently improved survivability of mice from radiation-induced death and reduced apoptotic cell death. We suggest that HAsPBPE nanoparticles are a promising candidate for the radio-sensitive delivery of CAPE.

## 4. Materials and Methods

### 4.1. Materials

HA was purchased from Lifecore Biomedical (Chaska, MN, USA) and its molecular weight is 7460 g/mol from the manufacturer’s data. In addition, 2,2′-thiobis(ethylamine) (TbEA) was purchased from the Tokyo Chemical Industry Co. Ltd., (Tokyo, Japan). Moreover, 4-(carboxymethyl)phenylboronic acid pinacol ester (PBPE), N-(3-dimethylaminopropyl)-N′-ethylcarbodiimide hydrochloride (EDAC), N-hydroxysuccinimide (NHS), 1-hydroxybenzotriazole hydrate (HOBt), CAPE, and 3-[4,5-dimethylthiazol-2-yl]-2,5-diphenyltetrazolium bromide (MTT) were purchased from Sigma-Aldrich Co. (St. Louis, MO, USA). Chlorin e6 (Ce6) was purchased from Frontier Sci. Co. (Logan, UT, USA). The dialysis membrane (molecular weight cut-off: 1000, 2000, and 8000 g/mol) was obtained from Spectrum Lab., Inc. (Rancho Dominguez, CA, USA). All the organic solvents such as dimethylsulfoxide (DMSO), ethyl alcohol, and chloroform were used as an HPLC grade.

### 4.2. Synthesis of HAsPBPE Conjugates

PBPE-TbEA conjugates: PBPE (262 mg, 1.0 mM) was dissolved in 6 mL of DMSO with an equivalent mole of EDAC and NHS. This was magnetically stirred for 3 h and then an equivalent mole of TbEA (120 mg, 1.0 mM) was added. This reaction was further stirred for more than 12 h. Following this, PBPE-TbEA conjugates were obtained by precipitation into ethanol and precipitates were washed with ethanol two times, filtered, and dried in a vacuum. The yield of PBPE-TbEA conjugates were higher than 89% (*w*/*w*). Yield = weight of PBPE-TbEA conjugates/(PBPE weight + TbEA weight) × 100.

HAsPBPE conjugates: HA (400 mg, 1 mM as a disaccharide units) was dissolved in 5 mL of deionized water. Then, EDAC (0.5 mM, 96 mg) and HOBt (68 mg, 0.5 mM) in 10 mL DMSO was added to this solution and then magnetically stirred for 3 h. Following this, 180 mg of PBPE in 10 mL DMSO was added to this solution and then this was reacted more than 24 h. Following this, reactants were introduced into the dialysis membrane (MWCO: 8000 g/mol) and then dialyzed against deionized water for more than 2 days. To prevent the saturation of organic solvent, deionized water was exchanged every 3 h intervals. After that, the aqueous solution was lyophilized for more than 2 days. The final yield of HAsPBPE conjugates was higher than 94% (*w*/*w*). The yield of HAsPBPE conjugates = weight of HAsPBPE conjugates/(weight of PBPE-TBEA conjugates weight + HA weight) × 100.

### 4.3. Characterization of Conjugates

The synthesis of HAsPBPE conjugates was confirmed with the ^1^H 500 MHz superconducting Fourier transform-nuclear magnetic resonance (NMR) spectroscopy (Varian Unity Inova; Varian Inc., Santa Clara, CA, USA).

The Fourier transform-infrared (FT-IR) spectroscopy (FT-IR 8700; Shimadzu, Osaka, Japan) was also used to confirm the synthesis of conjugates.

### 4.4. Preparation of Nanoparticles

HAsPBPE conjugates (20 mg) were dissolved in 3 mL DMSO/water mixtures (2.5/0.5, *v*/*v*) and CAPE (2 or 4 mg) dissolved in 1.0 mL of DMSO was mixed. This solution was magnetically stirred for 30 min. This solution was dropped into 10 mL of deionized water and then this solution was introduced into the dialysis membrane (MWCO: 2000 Da) and then dialyzed against deionized water for 24 h. To remove the organic solvent and unloaded drug, water was exchanged every 2~3 h intervals. Following this, the resulting nanoparticle solution was lyophilized or used for analysis. Empty nanoparticles were also prepared in a similar manner to the above method, except CAPE.

To measure drug contents, the UV spectrophotometer (Shimadzu UV-1601 spectrophotometer, Shimadzu Co. Ltd., Kyoto, Japan) was used as follows: Lyophilized nanoparticles (5 mg) dissolved in 10 mL of DMSO was diluted with DMSO. This solution was used to measure absorption at 336 nm. The same weight of empty nanoparticles was used as a blank test. Contents of CAPE in the nanoparticle were calculated as follows: Drug contents = (weight of CAPE/weight of nanoparticles) × 100.

### 4.5. Characterization of Nanoparticles

The morphology of CAPE-incorporated HAsPBPE nanoparticles was observed with TEM (H-7600, Hitachi Instruments Ltd., Tokyo, Japan). One drop of CAPE-incorporated HAsPBPE nanoparticles was placed onto the carbon film coated copper grid followed with drying in room temperature. This was used to observe the nanoparticle morphology at 80 kV.

The particle size distribution of nanoparticles (0.2%, *w*/*v* based on polymer weight) was analyzed with Nano-ZS (Malvern, Worcestershire, UK). Nanoparticles in deionized water were used to measure the particle size at room temperature.

For fluorescence emission spectra, CAPE-incorporated nanoparticles in phosphate buffered saline (PBS, pH 7.4, 0.01 M) were measured with the fluorescence spectrophotometer (RF-6000, Fluorescence fluorospectrophotometer, Shimadzu Co. Ltd., Kyoto, Japan) between 300 and 500 nm (excitation wavelength: 336 nm).

### 4.6. Drug Release Study

The release of CAPE from the nanoparticles was studied in vitro. The CAPE-incorporated nanoparticles (5 mg) were reconstituted in 5 mL of PBS (0.01 M, pH 7.4) and introduced into the dialysis tube (MWCO: 8000 g/mol). Following this, the mixture was put into 45 mL PBS in a 50 mL falcon tube and then agitated with a shaking incubator (100 rpm) at 37 °C. At predetermined time intervals, all of the media were taken to measure the released CAPE from the nanoparticles and replaced with fresh media to prevent saturation of CAPE in the media. The CAPE concentration was measured with the UV-spectrophotometer (Shimadzu UV spectrophotometer 1800; Shimadzu Company Co. Ltd., Kyoto, Japan) at 336 nm.

### 4.7. Cell Culture

L929 mouse fibroblast cells obtained from the Korean Cell Line Bank (KCLB, Korean Cell Line Bank Co. Ltd., Seoul, Korea) were cultured under 5% CO_2_ incubator at 37 °C. L929 cells were maintained and sub-cultured with RPMI1640 (Gibco, Grand Island, NY, USA) supplemented with 10% heat-inactivated fetal bovine serum (FBS) (Gibco^®^, Life Tech. Co., Grand Island, NY, USA) and 1% penicillin/streptomycin.

The cell viability assay was as follows: 2 × 10^4^ L929 cells seeded into 96-well plates were cultured in a 5% CO_2_ incubator at 37 °C overnight. CAPE, CAPE-incorporated nanoparticles or empty nanoparticles were treated to L929 cells. DMSO (final concentration: 0.5%, *v*/*v*) was used for the control treatment. CAPE dissolved in DMSO was diluted with serum-free media (final DMSO concentration: Less than 0.5%). CAPE-incorporated nanoparticles or empty nanoparticles in aqueous solution were sterilized with a 1.2 μm syringe filter. These were incubated in a 5% CO_2_ incubator for 4 h. Following this, cells were then exposed to hydrogen peroxide in serum-free media or irradiated with 6 MV photon beams using Clinac iX (Varian medical System, Palo Alto, CA, USA). For irradiation, the 96-well plate was placed from the source and exposed to 20 Gy radiation (45 cm from the surface, irradiation field: 40 × 40 cm). Following this, cells were incubated 1 day in a 5% CO_2_ incubator at 37 °C and then viability was tested with the MTT cell proliferation assay. One day later, the media were replaced with the serum-free media containing the MTT reagent (0.5 mg/mL) and then further incubated at 37 °C for 4 h. Then, the supernatants were removed and viable cells were dissolved in DMSO. The viability of L929 cells was evaluated with an Infinite M200 pro microplate reader (Tecan Trading AG, Mannedorf, Switzerland) at 570 nm.

### 4.8. Fluorescence Observation of Nanoparticle Uptake into Cells

To investigate the cellular uptake of nanoparticles, HAsPBPE conjugates (20 mg) that were dissolved in 3 mL DMSO/water mixtures (2.5/0.5, *v*/*v*) and Ce6 (2 mg) dissolved in 1.0 mL of DMSO were added. This solution was dropped into 5 mL of deionized water and then magnetically stirred for 30 min. Then, this solution was introduced into the dialysis membrane (MWCO: 2000 Da) and then dialyzed against deionized water for 24 h with an exchange of water at 2~3 h intervals. Following this, the resulting nanoparticle solutions were lyophilized or used for analysis. The Ce6 contents in the nanoparticles were measured with the Infinite M200 pro microplate reader (Tecan Trading AG, Mannedorf, Switzerland) at 407 nm of excitation wavelength and 664 emission wavelength. The contents of Ce6 in the nanoparticles were calculated as follows: Drug contents = (Weight of Ce6/weight of nanoparticles) × 100. The Ce6 contents in the nanoparticles were 8.6% (*w*/*w*).

The Ce6-incorporated nanoparticles (0.1 mg as HAsPBPE conjugates) in the serum-free RPMI1640 media were treated to the cells. After 4 h, the cells were washed with PBS and then observed with a fluorescence microscope (Eclipse 80i, Nikon, Tokyo, Japan).

### 4.9. ROS Assay

The cellular production of ROS was evaluated by the DCFH-DA method. L929 cells (2 × 10^4^ cells) in a 96-well plate that were treated with CAPE, empty nanoparticles or CAPE-incorporated nanoparticles for 4 h at 37 °C and then DCFH-DA (final concentration: 20 μM) in the phenol red-free RPMI media were added. These were irradiated as shown, in which cells were washed with PBS twice, added with 100 μL fresh phenol red free RPMI media, and irradiated at 664 nm (2.0 J/cm^2^). The ROS generation was evaluated by fluorescence intensity at an excitation wavelength of 485 nm and emission wavelength of 535 nm using Infinite M200 pro microplate reader.

### 4.10. In Vivo Radiation of Animal Model

The in vivo animal study was performed using BALb/C mouse (male, 5 weeks, 20 g). The treatment groups were divided into three groups: Control, injected with PBS; treatment with CAPE + empty nanoparticles, 10 mg/kg CAPE + 100 mg/mL empty nanoparticles; and CAPE-incorporated nanoparticles, 10 mg/kg as a CAPE dose. For the administration of CAPE, CAPE was dissolved in mixtures of ethyl alcohol and Cremophor EL (1/1, *v*/*v*) and then diluted with the PBS solution more than 10 times. For the nanoparticle treatment, nanoparticles were sterilized with a 1.2 μm syringe filter. All the treatments were administered intravenously (i.v.) with an injection volume of 100 μL via the tail vein of mice. Three hours later, mice were irradiated (irradiation dose: 7 Gy) using 6 MV photon beams using Clinac iX (Varian Medical System, Palo Alto, CA, USA) with a surface distance of 45 cm from the mice. For the observation of survivability of mice, mice were maintained under a controlled temperature and humidity. Feed and water were freely provided during the observation of survivability of mice. Some of the mice were sacrificed to observe the effect of irradiation against organs such as liver and spleen 1 day later from irradiation. Organs were isolated and immersed into the 4% paraformaldehyde solution. These were then fixed with 4% formamide, paraffin-embedded, and sliced for hematoxylin/eosin staining and terminal deoxynucleotidyl transferase dUTP nick end labeling (TUNEL) staining.

For histological analysis, the liver and spleen tissues were collected 3 h later from the irradiation. Liver and spleen tissues were fixed immediately with 10% neutral buffered formalin, then processed for paraffin embedding and sectioning. Staining for hematoxylin and eosin (H&E) was performed on a Leica auto strainer XL (Leica Biosystems, Germany). For the TUNEL assay, the TUNEL Assay kit-HRP-DAB (ab206386, Abcam, Cambridge, MA 02139-1517, USA) was performed following the manufacturer’s protocol.

### 4.11. Statistical Analysis

The statistical significance of the results were evaluated with the Student’s *t*-test using the SigmaPlot^®^ program.

## Figures and Tables

**Table 1:** Characterization of CAPE-incorporated nanoparticles.

Polymer/Drug Weight Ratio (mg/mg)	Drug Contents (%, *w/w*)		Average Particle Size (nm)
	Theoretical	Experimental	
20/0 20/2 20/4	- 9.1 16.7	- 7.5 12.3	64.8 ± 4.8 82.1 ± 9.6 101.3 ± 12.3

## Data Availability

Not applicable.

## Supplementary Materials

The following are available online at https://www.mdpi.com/article/10.3390/ijms22126347/s1.
