# Supplementary material for: Caffeic Acid Phenethyl Ester-Incorporated Radio-Sensitive Nanoparticles of Phenylboronic Acid Pinacol Ester-Conjugated Hyaluronic Acid for Application in Radioprotection
Source: Int J Mol Sci. 2021 Jun 14;22(12):6347. doi: 10.3390/ijms22126347 (PMC8231778; doi:10.3390/ijms22126347)
Supplement: Supplementary file 1 [file ijms-22-06347-s001.zip › ijms-1232211-supplementary.pdf]

Supporting materials

Materials and methods

Histological analysis

Spleen tissues treated for TUNEL assay (TUNEL Assay kit-HRP-DAB (ab206386, Abcam, USA)) was used for capture of apoptotic cells (300 μm × 300 μm). In spleen tissue sections, ten areas were randomly captured and counted apoptotic cells (dark spot).

Results

As shown in Figure s1, ten areas (300 μm × 300 μm) in spleen tissues were randomly selected for analysis of apoptotic cells and then counted apoptotic cells (dark spot). Figure s2 shows the selected areas for analysis of apoptotic cells. As shown in figure s2, apoptotic cells were significantly decreased when CAPE or CAPE-incorporated nanoparticles were treate

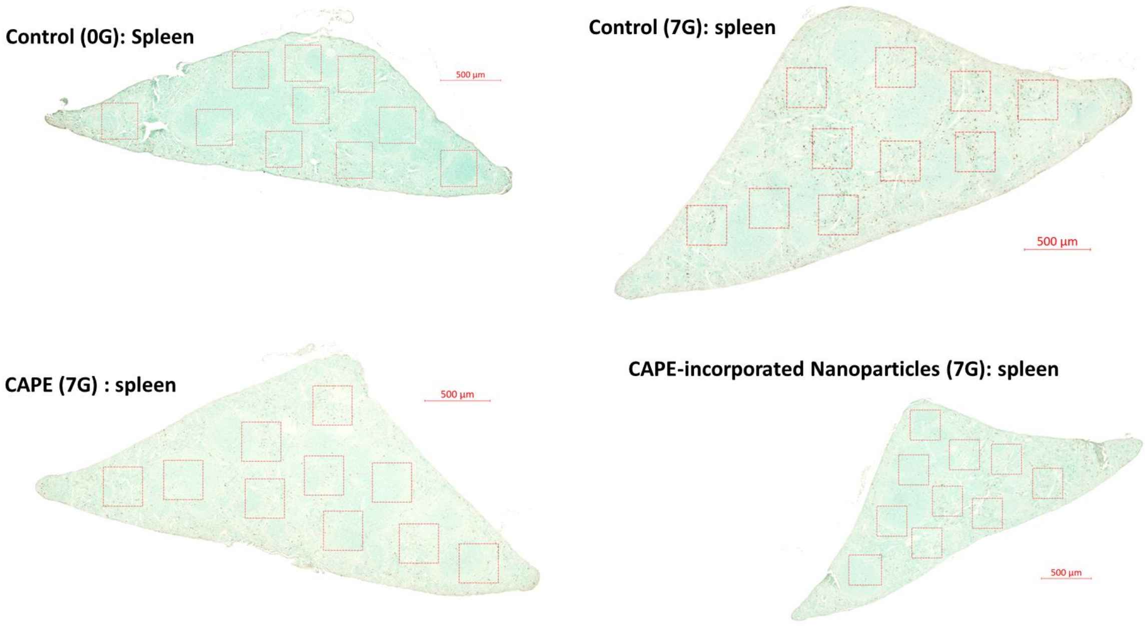

Figure S1. Spleen tissue sections for analysis of apoptotic cell death.

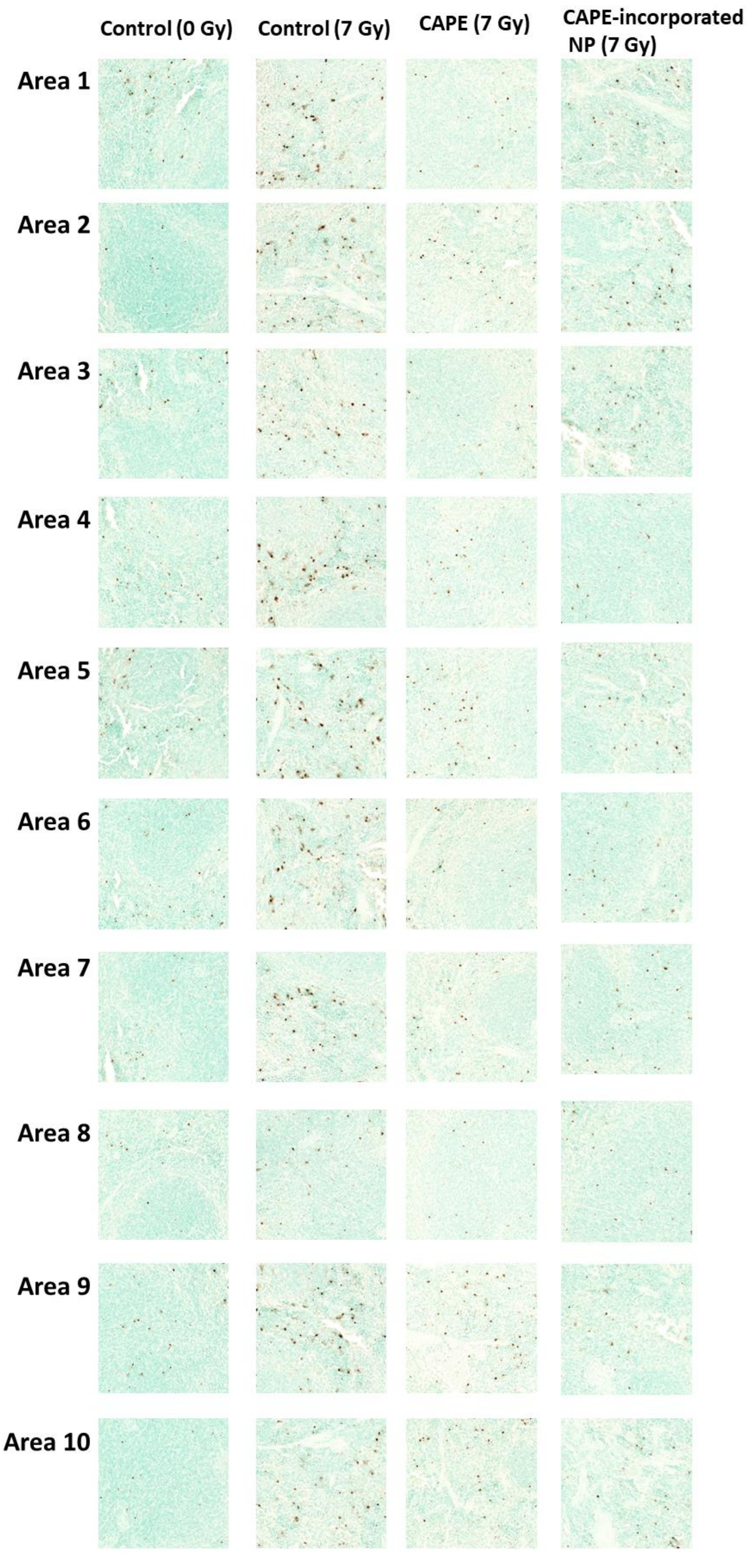

Figure S2. Ten areas for analysis of apoptotic cell death.
